# An Analysis of Biomechanical and Physiological Changes During Abdominal Wall Reconstruction

**DOI:** 10.3389/jaws.2026.16437

**Published:** 2026-03-26

**Authors:** S. G. Parker, A. L. A. Bloemendaal, T. Pampiglione, S. Mallett, S. Halligan, J. McCullough, A. C. J. Windsor, A. A. O. Plumb

**Affiliations:** 1 The Abdominal Wall Unit, Croydon University Hospital, London, United Kingdom; 2 Department of Surgery, Reinier de Graaf Gasthuis, Delft, Netherlands; 3 General Surgery Department, University College London Hospitals, London, United Kingdom; 4 Centre for Medical Imaging, University College London, London, United Kingdom; 5 Department of Surgery, The Princess Grace Hospital, London, United Kingdom; 6 Department of Radiology, University College London Hospitals, London, United Kingdom

**Keywords:** abdominal wall reconstruction, intra-abdominal pressure, lung compliance, tension, tension-free

## Abstract

**Introduction:**

Abdominal wall reconstruction (AWR) creates biomechanical and physiological changes, impacting the respiratory system. We assessed how dynamic lung compliance (LC) changed in response to intra-abdominal pressure (IAP) and closure forces. Secondarily, we investigated if patient, radiological, or biomechanical factors were identifying predictors of physiological changes.

**Materials and methods:**

We performed a prospective observational study in patients undergoing complex ventral hernia repair. LC was measured during 3 intra-operative stages after full muscle relaxation. Primary outcome was change in LC between completion of adhesiolysis and closure. Additionally, we measured force and distance required for midline closure.

**Results:**

Nineteen patients (median age 63) underwent AWR. Median hernia volume was 437 cm^3^ (IQR, 233–1,608). Mean LC change was −6.8 mL/cmH_2_O (±6 SD). Mean IAP increase was 3 mmHg (±1.7 SD). LC reduced and IAP increased between adhesiolysis and skin closure, significantly (*P* < 0.001). Increased midline closure distance was positively associated with increased closure force (*P* = 0.03, 0.12N, 95% CI 0.21–4.0). There was no evidence that increased closure force reduced LC or raised final IAP. Preoperative FEV1 and BMI were associated with reduced final LC (*P* = 0.05, 6.23L 95% CI 0.05 to 12.4; *P* = 0.03, −0.74 kg/m2, 95% CI −1.4 to −0.07). There was no evidence of an association between radiological measurements and change of LC or IAP.

**Discussion:**

Reduced LC is positively associated with increased IAP. However, there is no evidence increased closure forces affect final LC or IAP. Pre-operative optimisation of BMI or pre-operative FEV1 may have more impact than hernia morphology on LC reduction.

## Introduction

Ventral hernia (VH) rates are increasing [1, 2]. Combined with an aging and increasingly obese population, surgical complexity and risk of complications rise significantly [3–6].

Although a “tension-free” hernia repair is advocated, little work has quantified this directly. Additionally, this terminology derives mainly from inguinal hernia repair [7]. Direct fascial closure avoids a bridging mesh; the latter being associated with higher recurrence rates [8] but will inevitably lead to tension on the anterior fascia, even more so in large and complex cases. Remarkably little is known about the relation between force needed to close the anterior fascia, the intra-abdominal pressure, and postoperative results and complications. Miller et al. have performed studies showing no relationship between force needed and 30-day postoperative outcome, but no long-term results have been published yet [9]. The few existing reports on the subject have all been in open surgery, whereas hernia surgery is being performed increasingly with laparoscopic and/or robotic assistance. To this end a minimally invasive tensiometry is being investigated [10].

Post-operative respiratory complications are reported in approximately 15% of VH repairs, which necessitates re-intubation in 4%–5% [11, 12]. This may lead to increasing length of hospitalisation, requiring discharge to rehabilitation facilities, mortality, and increased costs [13]. However, no studies directly measure the relationship between biomechanical closure forces and physiological respiratory changes that occur during AWR.

Biomechanically, the abdomen and thorax may be considered one compartment, a concept initially proposed by Rives and Trivellini, who advocated avoiding intra-abdominal hypertension to preserve respiratory function [14, 15]. Factors influencing pressure within this unified compartment are an increased internal pressure with a reduced compartment volume, or a reduced thoracic and/or abdominal wall compliance. In AWR, reduced abdominal volume contingent on fascial closure typically elevates intra-abdominal pressure (IAP). Consequently, if the abdominal and thoracic compartments are unified, thoracic pressure will also rise, compromising respiratory function.

We aimed to directly quantify the biomechanical and physiological changes during AWR. We investigated if varying biomechanical forces during the perioperative period affect intra- and post-operative pulmonary function, and to what degree. We hypothesised that increased closure forces or raised IAP will affect dynamic lung compliance (LC) negatively. Secondarily, we hypothesised that pre-operative hernia morphology, derived from cross-sectional imagining, may identify predictors of closure forces and thus peri-operative lung physiology.

## Materials and Methods

### Participants and Recruitment

A single arm prospective observational study was designed to measure physiological and biomechanical changes in patients undergoing surgery at a single institution with specialist AWR practice. Ethical approval was obtained and the study registered with ClinicalTrials.gov (ID: NCT03296475).

Patients were initially identified from their clinical details and pre-operative imaging, then approached for study enrolment and consent if they met the inclusion criteria. We included patients over 18 years old with midline hernia defects exceeding 5 cm wide or loss of domain (LOD) greater than 20% (Sabbagh methode) [16], both calculated using pre-operative computed tomography (CT) examinations. Hernias were classified as Ventral Hernia Working Group (VHWG) grade 2 or 3 [17]. We excluded patients with VHWG grade 4 hernias, defects not in the midline, AWR for malignant disease, patients with respiratory disease requiring steroids, on home oxygen therapy, immunosuppression, HIV, liver cirrhosis or American Society of Anaesthesiologists (ASA) grade 4 [18]. The method of AWR was at the discretion of the lead operating surgeon. Hernias could be closed with or without mesh augmentation and/or use of pre-operative botulinum toxin (Botox, Allergan, Dublin, Ireland).

### Outcomes

Consenting patients underwent three main categories of assessment for the study. Firstly, analysis of pre-operative cross-sectional imaging was performed to determine: (a) maximum axial distance between the medial recti; (b) axial and craniocaudal dimensions of the hernia defect; (c) abdominal wall and retroperitoneal fat thickness at 3 different craniocaudal levels; (d) the volume of the hernia sac and, (e) abdominal cavity volume. Volumes were calculated using an ellipsoid approximation method as previously described by Sabbagh et al [16].

Secondly, patients underwent pre- and intra-operative lung function testing. Pre-operative testing comprised spirometry performed by a clinical physiologist in the hospital lung function laboratory on the morning of surgery. We measured forced expiratory volume in 1 s (FEV1), total lung capacity, and tidal volume (ExpAir, Medisoft, Sorinnes, Belgium). Subsequently, we performed intra-operative physiological measurements at 3 specific stages, all after full muscle relaxation; (a) after induction prior to the commencement of surgery, (b) after full adhesiolysis had been completed, and (c) after skin closure. Physiological measurements recorded were dynamic lung compliance (LC), and IAP (via a bladder pressure transducer). Tidal volume was standardised to 6 mL/kg during measurement collation.

Thirdly, intra-operative biomechanical measurements were taken at maximum muscle relaxation, after full adhesiolysis and component separation (if required, at the judgement of the operating surgeon). The force, in Newtons, required to bring the anterior sheath to the midline was measured at 2 levels for each side: halfway between the xiphisternum and pubic symphysis; and at the maximal width of the hernia defect. Allis forceps were applied to the rectus sheath with gauze attached as a string. This was attached away from the patient to a Newton meter (BFG 50, Mecmesin Ltd., Slinford, UK) and the force required to bring the rectus sheath fascia into the midline measured. At the same points we measured distance, in cm, required to bring the sheath to the midline. All such biomechanical measurements were taken by the same investigator to ensure consistency.

Any local or general post-operative complication was documented. Hospital acquired pneumonia was defined as antibiotic requirement for clinical and/or radiological chest infection. Patients were followed for a minimum 1-year following AWR, either via face-to-face surgical clinics or telephone (due to COVID-19 pandemic).

The primary outcome was the change in dynamic lung compliance (LC) after full adhesiolysis was completed compared to after abdominal wall fascial and skin closure. Secondary outcomes were change in IAP, abdominal closure force, distance required to bring the sheath to the midline, and post-operative complications.

### Statistical Analysis

The sample size was determined by the primary outcome, change in LC between adhesiolysis and skin closure. Based on previous work by Pereira et al we anticipated a 3.5mL/cmH_2_O change in LC [19]. At 80% power with α = 0.05 we required a minimum sample size of 16 patients.

Tables of frequencies and percentages described participant characteristics, hernia morphology, intra-operative reconstructive techniques, and post-operative complications. Variables were described using mean (±standard deviation) if normally distributed or median (interquartile range [IQR]) if not. Scatter plots were used to display perioperative changes in LC and IAP. A 2-point scatter plot was used to show change in LC and IAP before and after abdominal closure, where measurements from the same patient are linked.

Differences in LC after adhesiolysis and after closure were compared using a paired t-test.

Relationships between intraoperative physiological measurements, radiological features, and patient factors were compared using univariable regression. Three multivariable regression models were created to find predictors of final LC. Statistical analysis was performed using Stata (Stata/IC 16.1, StataCorp, TX, USA).

## Results

### Patient Characteristics

After consent, 19 patients (7 male) were enrolled into the study; all underwent AWR and reached 1 year follow-up. Patient demographics and co-morbidities are summarised in Table 1. Median age was 63 years (range 39–73). Median body mass index (BMI) was 31 (IQR, 29–38). All participants had incisional VHs. Eleven (58%) had a history of post-operative wound infection after previous laparotomy or VH repair. Twelve (63%) had a history of multiple laparotomies. Seven (37%) had undergone a previous VH repair, with 2 (10%) having multiple repairs. Median hernia volume was 437 cm^3^ (IQR, 233–1,608) ranging from 22.12 to 6,417.3 cm^3^. Median defect width was 9.7 cm (IQR, 8–12) and median LOD was 8.1% (IQR, 2.1–16.9). Seven (37%) participants had pre-operative abdominal wall botulinum toxin injections.

**TABLE 1:** Patient characteristics, co-morbidities, surgical history, and pre-operative radiological findings.

Patient demographics	n = 19	
Sex M:F	7:12	​
​	Median (IQR)	Range
Age	63 (54-67)	39-73
BMI	31 (29-38)	26-46
​	n	%
Smoker	3	16
Ex-smoker	9	47
Never smoked	7	37
COPD (%)	1	5
Diabetes (%)	2	10
ASA		
Class II	10	52
Class III	9	48
Surgical History		
Incisional Hernia	19	100
Previous Wound Infection	11	58
Multiple Previous Laparotomies	12	63
Previous Repair	7	37
2 Previous Repairs	2	10
Pre-operative Radiology	Median (IQR)	Range
Hernia Volume (cm3)	437 (233–1608)	29.12–6417.3
Loss of Domain (%)	8.1 (2.1–17)	0-6–8.57
Separation of Recti (cm)	9.7 (8–12)	5.6–21.2
Length of Defect (cm)	11.9 (9.6–18.1)	4.4–21.3

### Clinical Procedures and Outcomes

VHs were repaired with various mesh positions; 6 (32%) intraperitoneal, 10 (53%) retrorectus, 1 bridging (5%) and 2 (10%) were closed primarily, without mesh (Table 2). Where mesh was used (17 patients), 7 (41%) had biologic, 9 (53%) biosynthetic, and 1 (6%) synthetic. There was no mortality. Five (26%) patients developed superficial wound dehiscence, 5 (26%) developed a seroma, and 1 (5%) developed a haematoma requiring surgical evacuation. Four (21%) patients developed post-operative pneumonia, and 1 (5%) developed a deep vein thrombosis (Table 3). Two (10%) patients required long-term negative pressure dressings, 1 of whom required this for 9 months. Ultimately, all wounds were closed, without hernia recurrence. Median follow-up was 21 months (IQR, 17.7–28.1).

**TABLE 2:** Surgical techniques used.

Surgical Techniques	n = 19	%
Pre-op Botox	7	37
Mesh Lie		
Intraperitoneal	6	32
Retrorectus	10	53
Bridging	1	5
Suture Repair	2	10
Type of Mesh		
Biologic	7	37
Biosynthetic	9	48
Synthetic	1	5

**TABLE 3:** 90 Day post-operative complications.

Post-operative Complications	n	%
Mortality	0	0
Wound infection	5	26
Seroma	5	26
Haematoma	1	5
Superficial Wound Dehiscence	4	21
Hospital Acquired Pneumonia	4	21
Deep Vein Thrombosis	1	5

### Biomechanical Outcomes

**FIGURE 1:**
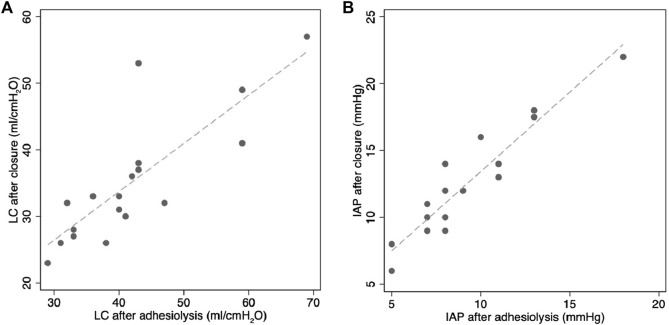
**(A)** LC and **(B)** IAP after full adhesiolysis and after skin closure (Table 4).

**FIGURE 2:**
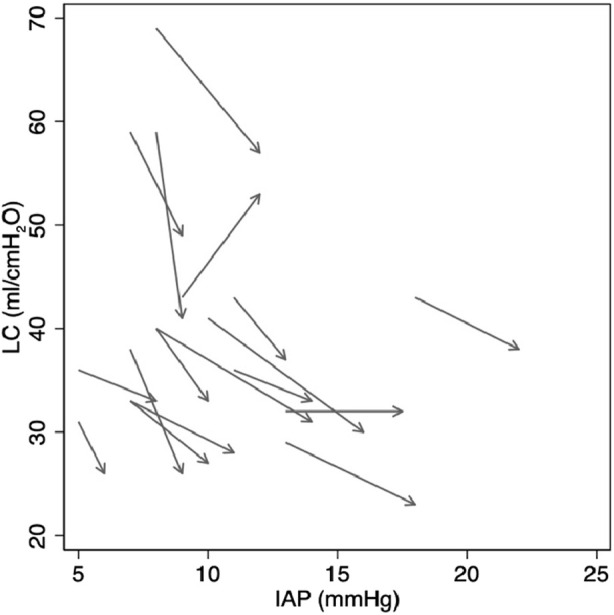
Linked individual patient data demonstrating change in LC and IAP from after adhesiolysis to after skin closure.

Concerning our primary outcome, we found LC dropped significantly between adhesiolysis and final skin closure. Mean change in LC was −6.8 mL/cmH_2_O (95% CI -9.7 to −3.9; *P* < 0.001) (Figure 1A; Table 4). IAP rose significantly from adhesiolysis to skin closure (mean increase of 3.3 mmHg, 95% CI 2.5 to 4.0; *P* < 0.001) (Figure 1B; Table 4). Figure 2 shows individual patient data demonstrating a rise in IAP and reduction in LC between adhesiolysis to skin closure in all but one case (where LC rose as IAP increased).

**TABLE 4:** Univariable analysis comparing physiological measurements after adhesiolysis and after fascial closure *denotes statistical significance.

Univariable Regression Analysis	After adhesiolysis	After fascial closure	Coeff.	*P*	*95% CI*
LC (ml/cmH2O) (mean, SD)	41.8 ( ± 10.5)	35 ( ± 9.3)	0.73	<0.001*	0.47 to 0.98
IAP (mmHg) (mean, SD)	9.1( ± 3.3)	12.3( ± 4.2)	1.18	<0.001*	0.95 to 1.42

**FIGURE 3:**
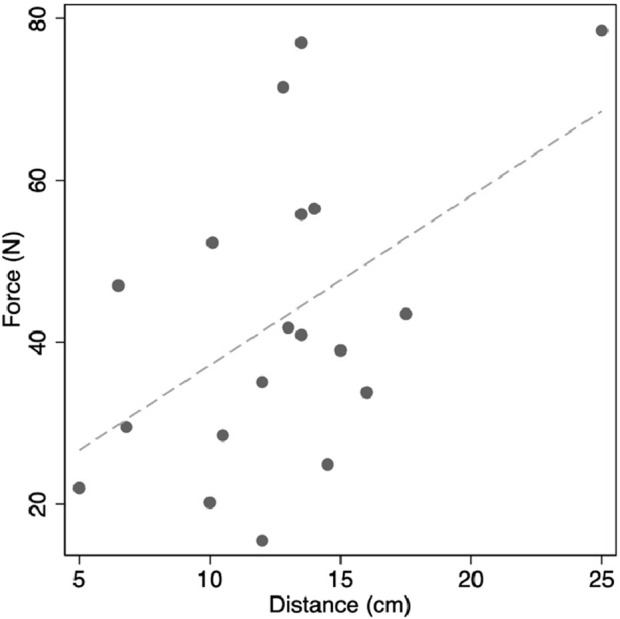
Positive association between fascial closure force and distance.

**FIGURE 4:**
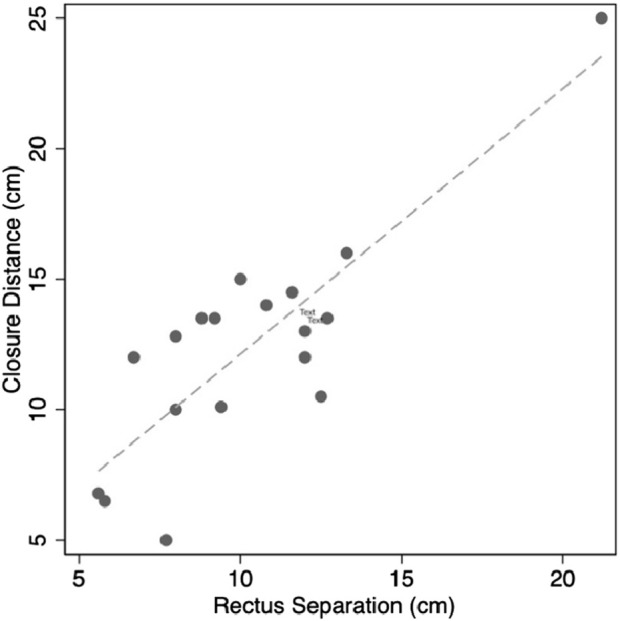
Positive association between radiological rectus separation and closure distance.

**FIGURE 5:**
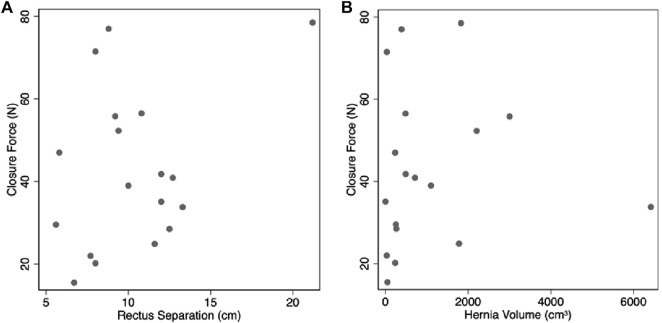
No evidence for an association between closure force and radiological. **(A)** rectus separation. **(B)** hernia volume.

After adhesiolysis, the median total distance (both sides combined), required to bring the fascia to the midline was 13 cm (IQR, 10.3 to 14.3, range 5–25 cm). Median combined force for both sides required to bring the fascia to the midline was 21N (IQR, 17.7 to 28.1, range 15.5–78.5N). There was no evidence for a relationship between the force or distance required to close and IAP (Table 5). There was a significant positive association between force required to close and the closure distance to the midline, with 1 cm extra closure distance requiring an extra 2N force (*P* = 0.03, Figure 3). The distance required to close and the separation of rectus muscles on pre-operative imaging had a significant positive association (*P* < 0.001, Figure 4). The force required to close was not related to the radiological rectus separation nor hernia volume (Figures 5A,B). Additionally, there was no association found between the force of closure and LOD or BMI.

**TABLE 5:** Univariable regression predicting final IAP and closure force *denotes statistical significance.

Univariable regression predicting final IAP			
​	Coeff.	P	(95% CI)
Combined closure distance (cm)	−0.07	0.74	(−0.52 to 0.38)
Combined closure force (N)	0.02	0.80	(−0.1 to 13.3)

Univariable regression predicting combined closure force (N)			
​	Coeff.	P	(95% CI)
Combined closure distance (cm)	2.1	0.03*	(0.21 to 3.98)
Separation of Recti (cm)	1.8	0.17	(-0.83 to 4.45)
Hernia Volume (cm3)	<0.001	0.78	(-0.006 to 0.007)
Loss of Domain (%)	0.15	0.53	(-0.34 to 0.63)
BMI (kg/m2)	1.17	0.14	(-0.41 to 2.76)

**FIGURE 6:**
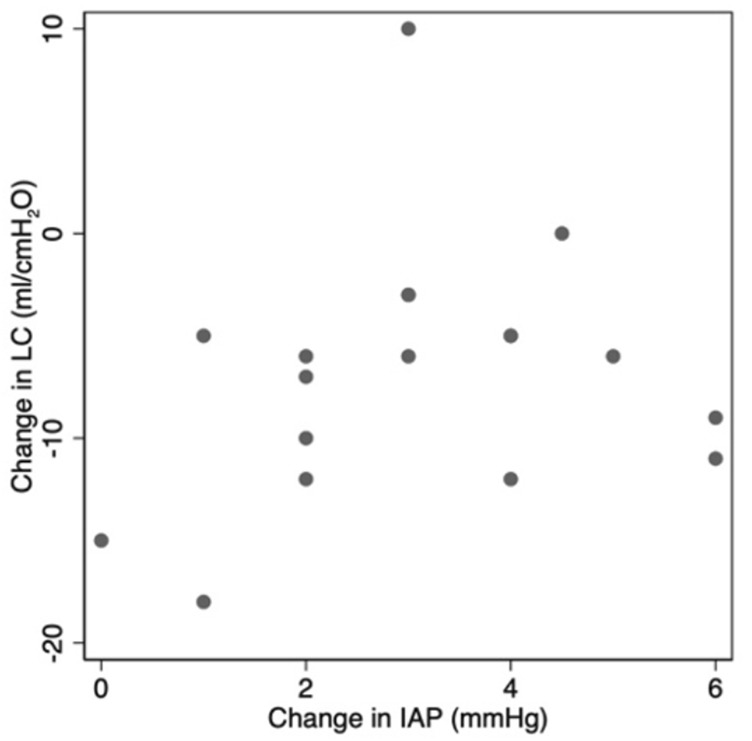
No evidence for an association between total change in IAP and LC from adhesiolysis to closure.

Three multivariable linear regression models were developed to identify predictors of final LC from pre-operative patient factors, radiological, and intraoperative findings (Table 6). These demonstrated that preoperative FEV1 and high BMI were significant predictors of final LC, with on average each increased unit of preop FEV1 increasing final LC by 6.23 (95% CI 0.05–12.4) and each increased unit of BMI decreasing final LC by 0.74 (95% CI 0.07–1.4). However, there was no evidence to show that combined force or distance required to close were associated with final LC, nor were radiological rectus separation, hernia volume or LOD (Table 6). A greater total increase in IAP was not associated with a larger decrease in LC (Figure 6).

**TABLE 6:** Three multivariable regression models predicting final LC from preoperative patient, radiological, and intraoperative measurements. *denotes statistical significance.

Multivariable regression models predicting final LC	Coeff.	*P*	(95% CI)
Patient Factors			
Preop. FEV1	6.23	0.048*	(0.05 to 12.4)
BMI (kg/m^2^)	−0.74	0.03*	(−1.4 to −0.07)
Radiological Factors			
Separation of Recti (cm)	−0.18	0.81	(−1.76 -to 1.39)
Hernia Volume (cm^3^)	<0.001	0.98	(−0.09 to 0.09)
Loss of Domain (%)	−0.65	0.83	(−0.72 to 0.59)
Intraoperative factors			
LC after adhesiolysis (ml/cmH_2_O)	0.77	<0.001*	(0.46 to 1.07)
Combined Closure Force (N)	0.08	0.4	(−0.12 to 0.28)
Combined Closure Distance (cm)	−0.27	0.48	(−1.06 to 0.52)
Change in IAP (mmHg)	0.39	0.67	(−1.53 to 2.3)

Exploratory analyses examined potential associations between biomechanical changes and post -op complications. We found no evidence of associations, although due to small numbers of participants (and so low numbers of complications, Table 3) these analyses had low power to detect any.

## Discussion

As surgeons perform increasingly complex abdominal wall reconstructions, it is desirable to know how much tension is permissible while avoiding respiratory complications and hernia recurrence. This study was designed to evaluate any relationship between the biomechanical and physiological changes that occur during complex AWR. We used 2 main approaches: Firstly, to identify whether LC declines significantly as IAP increases; and, secondly, to investigate how the force required to close the abdominal fascia affects LC and/or IAP. We found that LC decreased significantly after abdominal wall closure, which was negatively associated with IAP (Figure 2). However, we did not find that a greater total IAP increase was associated with further LC reduction (Figure 6). We found no evidence that increased fascial closure force either reduces LC or increases IAP significantly. Our results, in this series of patients, suggest that pre-operative respiratory function and obesity are better predictors of reduced post-operative lung compliance than intra-operative abdominal closure forces or pre-procedural radiological findings.

Our work extends that of Gaidukov et al., who found that increased IAP negatively impacts respiratory function with changes persisting after surgery [20]. It is a longstanding mantra that intra- and post-operative elevated IAP affects respiratory function negatively. However, little work has quantified this for AWR, with most research undertaken in a critical care setting where intra-abdominal hypertension is often secondary, e.g., as a response to alternate insult such as cardiac or hepatic failure [21, 22]. Typically, in this setting, raised IAP is caused by increased abdominal compartment content volume, due to oedema or ascites, rather than boundary restriction [23]. Additionally, in critical care these changes often occur gradually, in contrast to AWR where increased pressure occurs acutely on closure. Due to the nature of these complex operations, often combined with other procedures, intra-abdominal hypertension can be exacerbated further by visceral oedema and/or post-surgical inflammation.

We found increased abdominal closure forces did not increase IAP or reduce LC significantly. This probably reflects modern tension-reducing procedures, such as pre-operative Botox, component separation and transversus abdominus release, which aim to reduce tension. Tenzel et al. found that abdominal wall tension decreased after posterior component separation [24]. Therefore, given that these techniques are associated with relatively low tension, increased closure forces may be insufficient to affect IAP or LC significantly. It is possible that tension-relieving techniques are providing compensation, which forces must be sufficient to surpass before physiological changes occur. It may be the tension required to affect IAP or LC exceeds that acceptable for a surgically safe repair. Furthermore, subjective tension assessment performed by surgeons when closing fascia is unlikely to reflect true tension reliably. Hope et al. found that surgeons’ tension estimates did not agree with objective measurements using a tensiometer [25].

Radiological measurements did not show evidence of being predictors for decreased LC or increased closure forces, perhaps because these are obtained prior to component separation and/or botulinum toxin injections, interventions that mitigate against high closure forces and worsening LC. In particular, there was no evidence of an association between radiological rectus separation and closure force. However, radiological and intra-operative closure distances did have a significant positive association (Figure 4). As intra-operative closure distance and force were also significantly positively associated (Figure 3), we expect an appropriately powered study would demonstrate that pre-operative radiological measurements could identify predictors of intra-operative closure force. Doing so would assist pre-operative surgical strategy.

Our study has some important limitations. The study was powered to investigate change in LC rather than closure forces, post-operative complications, or hard endpoints such as recurrence, so our findings related to these other factors should be interpreted with caution. Increasing obesity may inflate the force measurements since the total weight of the abdominal wall is additionally transferred to the Newton meter. We encountered no recurrence, despite mean follow-up exceeding 12 months. Later recurrences will not be captured.

We conclude that reduced lung compliance occurs with increased IAP. However, raised IAP during and after AWR is multifactorial, and cannot be explained simply by either intra-operative closure force, closure distance, pre-operative hernia dimensions or LOD. Predictors of post-operative deterioration in lung compliance appear to be more general patient factors, pre-operative BMI and FEV1, rather than hernia-specific factors, including radiological measurements. This implies that respiratory complications following AWR are most likely to be diminished by pre-operative optimisation, rather than further reductions in closure force. Further research in larger cohorts is required to further assess the relationship between necessary closure force and short-term and long-term postoperative results, both in open and minimally invasive abdominal wall surgery.

## Data Availability

The raw data supporting the conclusions of this article will be made available by the authors, without undue reservation.
